# Poly[diaqua­(μ-oxalato)(μ-2-oxidopyridinium-3-carboxyl­ato)lanthanum(III)]

**DOI:** 10.1107/S1600536809018194

**Published:** 2009-05-23

**Authors:** Zhen Hu, Zhi-Bo Zhu

**Affiliations:** a Academic Affairs Division, Southern Medical University, Guangzhou, Guangdong 510515, People’s Republic of China; b School of Pharmaceutical Sciences, Southern Medical University, Guangzhou, Guangdong 510515, People’s Republic of China

## Abstract

In the title complex, [La(C_6_H_4_NO_3_)(C_2_O_4_)(H_2_O)_2_]_*n*_, the La^III^ ion is coordinated by eight O atoms from two 2-oxido­pyridinium-3-carboxyl­ate ligands, two oxalate ligands and two water mol­ecules in a distorted bicapped square-anti­prismatic geometry. The carboxyl­ate groups link adjacent La^III^ ions, forming two-dimensional layers that are further linked by N—H⋯O and O—H⋯O hydrogen bonds.

## Related literature

For related structures, see: Huang *et al.* (2009 ▶); Xu *et al.* (2009 ▶).
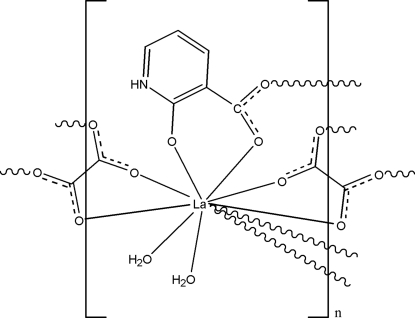


## Experimental

### 

#### Crystal data

[La(C_6_H_4_NO_3_)(C_2_O_4_)(H_2_O)_2_]
*M* *_r_* = 401.06
Triclinic, 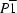

*a* = 8.0856 (18) Å
*b* = 8.5493 (19) Å
*c* = 9.388 (3) Å
α = 109.281 (3)°
β = 104.702 (3)°
γ = 104.940 (2)°
*V* = 549.5 (2) Å^3^
*Z* = 2
Mo *K*α radiation
μ = 3.93 mm^−1^
*T* = 293 K
0.20 × 0.18 × 0.17 mm

#### Data collection

Bruker APEXII CCD diffractometer
Absorption correction: multi-scan (*SADABS*; Sheldrick, 2003 ▶) *T*_min_ = 0.460, *T*_max_ = 0.512
2843 measured reflections
1946 independent reflections
1870 reflections with *I* > 2σ(*I*)
*R*_int_ = 0.020

#### Refinement

*R*[*F* ^2^ > 2σ(*F* ^2^)] = 0.025
*wR*(*F* ^2^) = 0.069
*S* = 1.10
1946 reflections
172 parameters
H-atom parameters constrained
Δρ_max_ = 0.92 e Å^−3^
Δρ_min_ = −1.37 e Å^−3^

### 

Data collection: *APEX2* (Bruker, 2004 ▶); cell refinement: *SAINT* (Bruker, 2004 ▶); data reduction: *SAINT*; program(s) used to solve structure: *SHELXS97* (Sheldrick, 2008 ▶); program(s) used to refine structure: *SHELXL97* (Sheldrick, 2008 ▶); molecular graphics: *SHELXTL* (Sheldrick, 2008 ▶); software used to prepare material for publication: *SHELXTL*.

## Supplementary Material

Crystal structure: contains datablocks I, global. DOI: 10.1107/S1600536809018194/bi2356sup1.cif

Structure factors: contains datablocks I. DOI: 10.1107/S1600536809018194/bi2356Isup2.hkl

Additional supplementary materials:  crystallographic information; 3D view; checkCIF report

## Figures and Tables

**Table 1:** Hydrogen-bond geometry (Å, °)

*D*—H⋯*A*	*D*—H	H⋯*A*	*D*⋯*A*	*D*—H⋯*A*
N1—H1*A*⋯O4^i^	0.86	1.96	2.789 (5)	162
O1*W*—H1*W*⋯O6^i^	0.85	2.01	2.805 (5)	155
O2*W*—H4*W*⋯O2*W*^ii^	0.85	2.00	2.853 (7)	180
O2*W*—H3*W*⋯O7^iii^	0.85	1.97	2.753 (5)	152

Symmetry codes: (i) 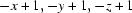; (ii) 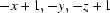; (iii) 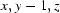.

**Fig. 1.**
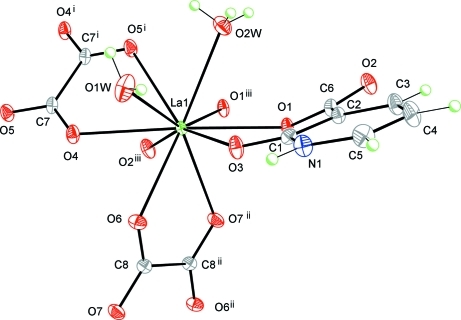
The molecular structure showing displacement ellipsoids at 30% probability for non-H atoms. Symmetry codes: (i) -x, -y, -z; (ii) -x, 1 - y, 1 - z; (iii) -x, -y, 1 - z. The H atoms on O2W are disordered.

**Fig. 2.**
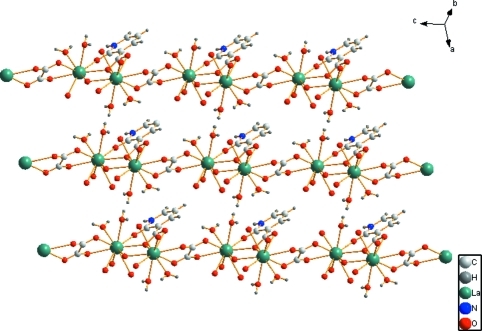
Packing diagram showing part of the 2-D layers (horizontal).

## supplementary crystallographic information

### Comment

Whereas a large number of metal derivatives of oxalic acid have been reported, there are few examples of metal derivatives of 2-oxynicotinic acid and oxalic acid: the crystal structures of praseodymium (Xu *et al.*, 2009) and dysprosium (Huang *et al.*, 2009) derivatives have been reported only. We report here a lanthanum(III) complex formed by reaction of lanthanum nitrate, 2-oxynicotinic acid and oxalic acid under hydrothermal conditions.

As illustrated in Fig. 1, each La^III^ centre adopts a distorted bicapped square-antiprismatic geometry, defined by eight O atoms from two 2-oxynicotinate ligands, two oxalate ligands, and two water molecules. The 2-oxynicotinate ligands and oxalate ligands link the La^III^ ions to form layers in the *bc* plane in which the shortest La···La separation is 4.429 (3) Å. These layers are connected through O—H···O and N—H···O hydrogen bonding (Table 1) involving 2-oxynicotinate ligands, oxalate ligands and the coordinating water molecules, forming a three-dimensional supramolecular network motif (Fig. 2).

### Experimental

A mixture of La_2_O_3_ (0.245 g, 0.75 mmol), 2-oxynicotinic acid (0.127 g, 1 mmol), oxalic acid (0.09 g, 1 mmol), water (10 ml) and HNO_3_ (0.024 g, 0.385 mmol) was stirred vigorously for 20 min then sealed in a Teflon-lined stainless-steel autoclave (20 ml capacity). The autoclave was heated and maintained at 433 K for 4 days, then cooled to room temperature at 5 K h^-1^ to yield colourless block crystals.

### Refinement

H atoms bound to C and N atoms were placed at calculated positions and refined as riding with N—H = 0.86 Å, C—H = 0.93 Å and with *U*_iso_(H) = 1.2 *U*_eq_(C/N). H atoms of the water molecules were tentatively located in difference Fourier maps and refined with distance restraints of O—H = 0.850 (1) Å and H···H = 1.350 (1) Å. In the final cycles of refinement, the O—H distances were normalized to 0.85 Å and the H atoms were refined as riding with *U*_iso_(H) = 1.5 *U*_eq_(O). Atom H4W forms a symmetrical H-bond about a centre of inversion and therefore is included with site occupancy factor 0.5. The alternative position H4W' points towards the centroid of an adjacent pyridyl ring.

### Crystal data

**Table:**

[La(C_6_H_4_NO_3_)(C_2_O_4_)(H_2_O)_2_]	*Z* = 2
*M**_r_* = 401.06	*F*(000) = 384
Triclinic, *P*1	*D*_x_ = 2.424 Mg m^−^^3^
Hall symbol: -P 1	Mo *K*α radiation, λ = 0.71073 Å
*a* = 8.0856 (18) Å	Cell parameters from 2827 reflections
*b* = 8.5493 (19) Å	θ = 2.5–28.3°
*c* = 9.388 (3) Å	µ = 3.93 mm^−^^1^
α = 109.281 (3)°	*T* = 293 K
β = 104.702 (3)°	Block, colourless
γ = 104.940 (2)°	0.20 × 0.18 × 0.17 mm
*V* = 549.5 (2) Å^3^	

### Data collection

**Table:**

Bruker APEXII CCD diffractometer	1946 independent reflections
Radiation source: fine-focus sealed tube	1870 reflections with *I* > 2σ(*I*)
graphite	*R*_int_ = 0.020
φ and ω scans	θ_max_ = 25.2°, θ_min_ = 2.5°
Absorption correction: multi-scan (*SADABS*; Sheldrick, 2003)	*h* = −7→9
*T*_min_ = 0.460, *T*_max_ = 0.512	*k* = −10→10
2843 measured reflections	*l* = −11→11

### Refinement

**Table:**

Refinement on *F*^2^	Primary atom site location: structure-invariant direct methods
Least-squares matrix: full	Secondary atom site location: difference Fourier map
*R*[*F*^2^ > 2σ(*F*^2^)] = 0.025	Hydrogen site location: inferred from neighbouring sites
*wR*(*F*^2^) = 0.069	H-atom parameters constrained
*S* = 1.10	*w* = 1/[σ^2^(*F*_o_^2^) + (0.0415*P*)^2^ + 0.4664*P*] where *P* = (*F*_o_^2^ + 2*F*_c_^2^)/3
1946 reflections	(Δ/σ)_max_ < 0.001
172 parameters	Δρ_max_ = 0.92 e Å^−^^3^
0 restraints	Δρ_min_ = −1.37 e Å^−^^3^

### Special details

**Table:**

Geometry. All e.s.d.'s (except the e.s.d. in the dihedral angle between two l.s. planes) are estimated using the full covariance matrix. The cell e.s.d.'s are taken into account individually in the estimation of e.s.d.'s in distances, angles and torsion angles; correlations between e.s.d.'s in cell parameters are only used when they are defined by crystal symmetry. An approximate (isotropic) treatment of cell e.s.d.'s is used for estimating e.s.d.'s involving l.s. planes.
Refinement. Refinement of *F*^2^ against ALL reflections. The weighted *R*-factor *wR* and goodness of fit *S* are based on *F*^2^, conventional *R*-factors *R* are based on *F*, with *F* set to zero for negative *F*^2^. The threshold expression of *F*^2^ > σ(*F*^2^) is used only for calculating *R*-factors(gt) *etc*. and is not relevant to the choice of reflections for refinement. *R*-factors based on *F*^2^ are statistically about twice as large as those based on *F*, and *R*- factors based on ALL data will be even larger.

### Fractional atomic coordinates and isotropic or equivalent isotropic displacement parameters (Å^2^)

**Table:**

	*x*	*y*	*z*	*U*_iso_*/*U*_eq_	Occ. (<1)
C1	0.5156 (6)	0.3528 (6)	0.7873 (5)	0.0193 (9)	
C2	0.4448 (6)	0.2225 (6)	0.8439 (5)	0.0190 (9)	
C3	0.5510 (7)	0.2276 (6)	0.9869 (5)	0.0278 (10)	
H3A	0.5043	0.1411	1.0209	0.033*	
C4	0.7265 (7)	0.3590 (7)	1.0820 (6)	0.0340 (12)	
H4A	0.7963	0.3617	1.1788	0.041*	
C5	0.7924 (6)	0.4829 (7)	1.0291 (6)	0.0310 (11)	
H5A	0.9085	0.5728	1.0910	0.037*	
C6	0.2549 (6)	0.0837 (6)	0.7555 (5)	0.0195 (9)	
C7	0.0124 (6)	0.0944 (6)	0.0035 (5)	0.0209 (9)	
C8	0.0714 (6)	0.5526 (5)	0.4754 (5)	0.0180 (8)	
La1	0.14649 (3)	0.16626 (3)	0.39929 (2)	0.01432 (11)	
N1	0.6911 (5)	0.4769 (5)	0.8873 (5)	0.0253 (8)	
H1A	0.7393	0.5561	0.8570	0.030*	
O1	0.1395 (4)	0.0964 (4)	0.6424 (3)	0.0215 (7)	
O2	0.2093 (4)	−0.0404 (4)	0.7976 (4)	0.0285 (7)	
O3	0.4372 (4)	0.3643 (4)	0.6604 (4)	0.0276 (7)	
O4	0.1045 (5)	0.2240 (4)	0.1390 (4)	0.0258 (7)	
O5	−0.0601 (5)	0.1020 (4)	−0.1273 (4)	0.0275 (7)	
O6	0.1897 (4)	0.4906 (4)	0.4461 (4)	0.0238 (7)	
O7	0.0581 (4)	0.6932 (4)	0.4686 (4)	0.0263 (7)	
O1W	0.4501 (5)	0.2684 (5)	0.3496 (4)	0.0400 (9)	
H1W	0.5512	0.3270	0.4309	0.060*	
H2W	0.4721	0.1911	0.2808	0.060*	
O2W	0.3055 (5)	−0.0633 (5)	0.4169 (4)	0.0329 (8)	
H3W	0.2604	−0.1491	0.4396	0.049*	
H4W	0.4214	−0.0254	0.4667	0.049*	0.50
H4W'	0.2871	−0.1202	0.3175	0.049*	0.50

### Atomic displacement parameters (Å^2^)

**Table:**

	*U*^11^	*U*^22^	*U*^33^	*U*^12^	*U*^13^	*U*^23^
C1	0.018 (2)	0.016 (2)	0.018 (2)	0.0023 (17)	0.0060 (17)	0.0040 (17)
C2	0.017 (2)	0.017 (2)	0.0160 (19)	0.0019 (17)	0.0018 (17)	0.0062 (17)
C3	0.027 (2)	0.023 (2)	0.024 (2)	0.002 (2)	0.000 (2)	0.012 (2)
C4	0.024 (3)	0.034 (3)	0.024 (2)	0.001 (2)	−0.011 (2)	0.012 (2)
C5	0.017 (2)	0.029 (3)	0.029 (2)	0.001 (2)	−0.0042 (19)	0.006 (2)
C6	0.016 (2)	0.020 (2)	0.016 (2)	−0.0004 (17)	0.0025 (17)	0.0074 (17)
C7	0.021 (2)	0.024 (2)	0.017 (2)	0.0035 (18)	0.0066 (18)	0.0103 (18)
C8	0.014 (2)	0.014 (2)	0.017 (2)	0.0005 (17)	−0.0001 (16)	0.0029 (17)
La1	0.01183 (15)	0.01383 (16)	0.01434 (15)	0.00163 (11)	0.00223 (11)	0.00694 (11)
N1	0.0186 (19)	0.022 (2)	0.027 (2)	−0.0013 (16)	0.0045 (16)	0.0110 (17)
O1	0.0154 (15)	0.0253 (17)	0.0172 (15)	0.0003 (13)	−0.0005 (12)	0.0118 (13)
O2	0.0215 (17)	0.0269 (18)	0.0325 (18)	0.0002 (14)	0.0028 (14)	0.0192 (15)
O3	0.0211 (17)	0.0312 (18)	0.0235 (16)	−0.0002 (14)	0.0014 (14)	0.0163 (14)
O4	0.0326 (19)	0.0190 (16)	0.0157 (15)	−0.0002 (14)	0.0040 (14)	0.0068 (13)
O5	0.0369 (19)	0.0238 (16)	0.0163 (15)	0.0085 (15)	0.0030 (14)	0.0093 (13)
O6	0.0193 (16)	0.0192 (16)	0.0336 (17)	0.0070 (13)	0.0107 (14)	0.0116 (14)
O7	0.0294 (18)	0.0209 (16)	0.0373 (18)	0.0110 (14)	0.0169 (15)	0.0177 (14)
O1W	0.0197 (18)	0.057 (2)	0.0307 (19)	0.0001 (17)	0.0090 (15)	0.0158 (18)
O2W	0.0285 (18)	0.040 (2)	0.043 (2)	0.0178 (16)	0.0155 (16)	0.0276 (17)

### Geometric parameters (Å, °)

**Table:**

C1—O3	1.248 (5)	La1—O6	2.574 (3)
C1—N1	1.382 (6)	La1—O5^i^	2.582 (3)
C1—C2	1.437 (6)	La1—O3	2.585 (3)
C2—C3	1.377 (6)	La1—O1W	2.598 (3)
C2—C6	1.489 (6)	La1—O4	2.606 (3)
C3—C4	1.395 (7)	La1—O7^ii^	2.608 (3)
C3—H3A	0.930	La1—O1^iii^	2.612 (3)
C4—C5	1.357 (7)	La1—O2W	2.634 (3)
C4—H4A	0.930	La1—O2^iii^	2.691 (3)
C5—N1	1.351 (6)	N1—H1A	0.860
C5—H5A	0.930	O1—La1^iii^	2.612 (3)
C6—O2	1.252 (5)	O2—La1^iii^	2.691 (3)
C6—O1	1.279 (5)	O5—La1^i^	2.582 (3)
C7—O4	1.250 (5)	O7—La1^ii^	2.608 (3)
C7—O5	1.251 (5)	O1W—H1W	0.850
C7—C7^i^	1.550 (9)	O1W—H2W	0.850
C8—O6	1.253 (5)	O2W—H3W	0.850
C8—O7	1.255 (5)	O2W—H4W	0.850
C8—C8^ii^	1.537 (8)	O2W—H4W'	0.850
La1—O1	2.553 (3)		
			
O3—C1—N1	118.0 (4)	O1—La1—O1^iii^	61.92 (11)
O3—C1—C2	127.4 (4)	O6—La1—O1^iii^	130.59 (10)
N1—C1—C2	114.6 (4)	O5^i^—La1—O1^iii^	70.65 (10)
C3—C2—C1	120.0 (4)	O3—La1—O1^iii^	127.57 (9)
C3—C2—C6	118.0 (4)	O1W—La1—O1^iii^	148.31 (12)
C1—C2—C6	121.9 (4)	O4—La1—O1^iii^	111.41 (10)
C2—C3—C4	121.8 (4)	O7^ii^—La1—O1^iii^	72.58 (10)
C2—C3—H3A	119.1	O1—La1—O2W	68.95 (10)
C4—C3—H3A	119.1	O6—La1—O2W	147.03 (10)
C5—C4—C3	118.1 (4)	O5^i^—La1—O2W	64.56 (10)
C5—C4—H4A	121.0	O3—La1—O2W	78.73 (11)
C3—C4—H4A	121.0	O1W—La1—O2W	72.87 (12)
N1—C5—C4	120.6 (4)	O4—La1—O2W	115.38 (10)
N1—C5—H5A	119.7	O7^ii^—La1—O2W	138.08 (10)
C4—C5—H5A	119.7	O1^iii^—La1—O2W	81.33 (10)
O2—C6—O1	121.1 (4)	O1—La1—O2^iii^	105.06 (9)
O2—C6—C2	119.0 (4)	O6—La1—O2^iii^	92.61 (10)
O1—C6—C2	119.8 (4)	O5^i^—La1—O2^iii^	66.21 (10)
O4—C7—O5	126.4 (4)	O3—La1—O2^iii^	157.15 (11)
O4—C7—C7^i^	116.9 (4)	O1W—La1—O2^iii^	131.48 (11)
O5—C7—C7^i^	116.7 (5)	O4—La1—O2^iii^	67.14 (10)
O6—C8—O7	126.1 (4)	O7^ii^—La1—O2^iii^	65.52 (10)
O6—C8—C8^ii^	117.3 (4)	O1^iii^—La1—O2^iii^	49.08 (9)
O7—C8—C8^ii^	116.6 (4)	O2W—La1—O2^iii^	118.71 (11)
O1—La1—O6	115.01 (10)	C5—N1—C1	124.9 (4)
O1—La1—O5^i^	116.73 (10)	C5—N1—H1A	117.6
O6—La1—O5^i^	127.50 (10)	C1—N1—H1A	117.6
O1—La1—O3	65.68 (10)	C6—O1—La1	135.9 (3)
O6—La1—O3	74.38 (10)	C6—O1—La1^iii^	96.3 (3)
O5^i^—La1—O3	136.55 (11)	La1—O1—La1^iii^	118.08 (11)
O1—La1—O1W	121.99 (10)	C6—O2—La1^iii^	93.3 (2)
O6—La1—O1W	78.71 (11)	C1—O3—La1	136.4 (3)
O5^i^—La1—O1W	81.66 (11)	C7—O4—La1	118.5 (3)
O3—La1—O1W	65.28 (11)	C7—O5—La1^i^	119.4 (3)
O1—La1—O4	172.10 (10)	C8—O6—La1	120.7 (3)
O6—La1—O4	65.38 (10)	C8—O7—La1^ii^	119.8 (3)
O5^i^—La1—O4	62.14 (10)	La1—O1W—H1W	118.9
O3—La1—O4	120.98 (10)	La1—O1W—H2W	118.3
O1W—La1—O4	65.90 (11)	H1W—O1W—H2W	105.2
O1—La1—O7^ii^	69.92 (10)	La1—O2W—H3W	122.1
O6—La1—O7^ii^	62.13 (9)	La1—O2W—H4W	119.6
O5^i^—La1—O7^ii^	131.12 (11)	H3W—O2W—H4W	105.2
O3—La1—O7^ii^	91.67 (11)	La1—O2W—H4W'	101.0
O1W—La1—O7^ii^	139.07 (11)	H3W—O2W—H4W'	100.6
O4—La1—O7^ii^	104.60 (10)	H4W—O2W—H4W'	105.2
			
O3—C1—C2—C3	179.2 (4)	N1—C1—O3—La1	168.2 (3)
N1—C1—C2—C3	−0.4 (6)	C2—C1—O3—La1	−11.5 (7)
O3—C1—C2—C6	−3.1 (7)	O1—La1—O3—C1	22.7 (4)
N1—C1—C2—C6	177.2 (4)	O6—La1—O3—C1	150.2 (4)
C1—C2—C3—C4	1.3 (7)	O5^i^—La1—O3—C1	−81.2 (4)
C6—C2—C3—C4	−176.5 (4)	O1W—La1—O3—C1	−125.3 (4)
C2—C3—C4—C5	−0.6 (8)	O4—La1—O3—C1	−162.1 (4)
C3—C4—C5—N1	−0.8 (8)	O7^ii^—La1—O3—C1	89.7 (4)
C3—C2—C6—O2	−11.8 (6)	O1^iii^—La1—O3—C1	20.6 (5)
C1—C2—C6—O2	170.5 (4)	O2W—La1—O3—C1	−49.1 (4)
C3—C2—C6—O1	165.6 (4)	O2^iii^—La1—O3—C1	92.9 (5)
C1—C2—C6—O1	−12.0 (6)	O5—C7—O4—La1	160.6 (4)
C4—C5—N1—C1	1.7 (7)	C7^i^—C7—O4—La1	−20.2 (6)
O3—C1—N1—C5	179.3 (4)	O6—La1—O4—C7	−157.7 (3)
C2—C1—N1—C5	−1.0 (6)	O5^i^—La1—O4—C7	20.9 (3)
O2—C6—O1—La1	−137.1 (4)	O3—La1—O4—C7	150.7 (3)
C2—C6—O1—La1	45.4 (6)	O1W—La1—O4—C7	114.1 (3)
O2—C6—O1—La1^iii^	5.4 (4)	O7^ii^—La1—O4—C7	−108.3 (3)
C2—C6—O1—La1^iii^	−172.0 (3)	O1^iii^—La1—O4—C7	−31.6 (4)
O6—La1—O1—C6	−98.9 (4)	O2W—La1—O4—C7	58.8 (3)
O5^i^—La1—O1—C6	90.3 (4)	O2^iii^—La1—O4—C7	−53.3 (3)
O3—La1—O1—C6	−41.3 (4)	O4—C7—O5—La1^i^	159.7 (4)
O1W—La1—O1—C6	−6.8 (4)	C7^i^—C7—O5—La1^i^	−19.6 (6)
O7^ii^—La1—O1—C6	−142.8 (4)	O7—C8—O6—La1	−165.0 (3)
O1^iii^—La1—O1—C6	136.7 (5)	C8^ii^—C8—O6—La1	15.1 (6)
O2W—La1—O1—C6	45.5 (4)	O1—La1—O6—C8	−63.1 (3)
O2^iii^—La1—O1—C6	160.9 (4)	O5^i^—La1—O6—C8	106.6 (3)
O6—La1—O1—La1^iii^	124.39 (13)	O3—La1—O6—C8	−116.0 (3)
O5^i^—La1—O1—La1^iii^	−46.43 (16)	O1W—La1—O6—C8	176.7 (3)
O3—La1—O1—La1^iii^	−178.07 (17)	O4—La1—O6—C8	108.2 (3)
O1W—La1—O1—La1^iii^	−143.50 (14)	O7^ii^—La1—O6—C8	−15.6 (3)
O7^ii^—La1—O1—La1^iii^	80.43 (14)	O1^iii^—La1—O6—C8	10.4 (4)
O1^iii^—La1—O1—La1^iii^	0.0	O2W—La1—O6—C8	−152.6 (3)
O2W—La1—O1—La1^iii^	−91.27 (15)	O2^iii^—La1—O6—C8	44.9 (3)
O2^iii^—La1—O1—La1^iii^	24.16 (15)	O6—C8—O7—La1^ii^	−165.3 (3)
O1—C6—O2—La1^iii^	−5.2 (4)	C8^ii^—C8—O7—La1^ii^	14.7 (6)
C2—C6—O2—La1^iii^	172.2 (3)		

Symmetry codes: (i) −*x*, −*y*, −*z*; (ii) −*x*, −*y*+1, −*z*+1; (iii) −*x*, −*y*, −*z*+1.

### Hydrogen-bond geometry (Å, °)

**Table:**

*D*—H···*A*	*D*—H	H···*A*	*D*···*A*	*D*—H···*A*
N1—H1A···O4^iv^	0.86	1.96	2.789 (5)	162
O1W—H1W···O6^iv^	0.85	2.01	2.805 (5)	155
O2W—H4W···O2W^v^	0.85	2.00	2.853 (7)	180
O2W—H3W···O7^vi^	0.85	1.97	2.753 (5)	152

Symmetry codes: (iv) −*x*+1, −*y*+1, −*z*+1; (v) −*x*+1, −*y*, −*z*+1; (vi) *x*, *y*−1, *z*.

## References

[bb1] Bruker (2004). *APEX2* and *SAINT* Bruker AXS Inc., Madison, Wisconsin, USA.

[bb2] Huang, C.-D., Huang, J.-X., Wu, Y.-Y., Lian, Y.-Y. & Zeng, R.-H. (2009). *Acta Cryst.* E**65**, m177–m178. 10.1107/S1600536809000580 PMC2968133 21581782

[bb3] Sheldrick, G. M. (2003). *SADABS* University of Göttingen, Germany.

[bb4] Sheldrick, G. M. (2008). *Acta Cryst.* A**64**, 112–122. 10.1107/S0108767307043930 18156677

[bb5] Xu, Y.-J., Yang, X.-X. & Zhao, H.-B. (2009). *Acta Cryst.* E**65**, m310. 10.1107/S160053680900542X PMC2968694 21582087

